# Point‐Guided Latent Diffusion Model for Novel View Synthesis in Laparoscopic Liver Surgery

**DOI:** 10.1049/htl2.70032

**Published:** 2025-11-18

**Authors:** Wenzhe Tang, Tao Chen, Yamid Espinel, Shahid Farid, Emmanuel BUC, Adrien Bartoli, Sharib Ali

**Affiliations:** ^1^ School of Computer Science, Faculty of Engineering and Physical Sciences University of Leeds Leeds UK; ^2^ Centre Hospitalier Universitaire de Clermont‐Ferrand Clermont‐Ferrand France; ^3^ Department of HPB and Transplant Surgery St. James's University Hospital Leeds UK; ^4^ Endoscopy and Computer Vision Group Université Clermont Auvergne Clermont‐Ferrand France

**Keywords:** computer vision, image reconstruction, liver, medical image processing

## Abstract

Despite recent progress in diffusion‐based video synthesis, synthesizing accurate novel views from sparse input frames in laparoscopic liver surgery remains challenging due to occlusions, complex shape of anatomical structures and limited field of views. We propose point‐guided latent diffusion model, specifically designed for generating high‐quality intermediate frames in laparoscopic liver surgery from only the first and last video frames. Our method leverages the powerful generative capability of latent diffusion models combined with geometric cues from 3D point clouds reconstructed via dense stereo matching. To robustly handle occlusions and shape deformation, we use an adaptive camera trajectory planning strategy based on next‐best‐view algorithms. Furthermore, we introduce a spatial‐transformer enhanced decoder to effectively preserve detailed anatomical features from reference frames and minimize visual artefacts in generated views. Extensive experiments on the clinically relevant P2ILF challenge dataset validate our method's effectiveness and superior performance in producing visually coherent and structurally accurate novel views, highlighting its ability for enhancing the quality of surgical scene reconstruction.

## Graphical abstract

This approach enables novel view synthesis from sparse laparoscopic inputs, reconstructing anatomically consistent views of regions not directly captured. It improves intra‐operative situational awareness, supports surgical training by generating multiple perspectives from limited recordings. The evaluation results demonstrate that the proposed approach leads to the performance of novel synthesis compared with SVD, CF‐3DGS and ViewCrafter in terms of PNSR, SSIM and LPIPS metrics.

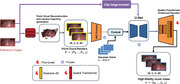

## 1 Introduction

Laparoscopic liver surgery demands precise spatial perception to ensure safe and effective resection around critical anatomical structures. Nevertheless, intraoperative visualization is inherently constrained by the limited viewpoint of a single laparoscopic camera [1]. The acquisition of high‐resolution laparoscopic video is further restricted by storage and transmission constraints. For instance, a single 4K video stream at 60 frames‐per‐second (fps) can generate hundreds of gigabytes of data during one operation, rendering the storage or transmission of complete raw streams impractical [2]. Consequently, clinical practice often relies on high‐compression or reduced‐resolution storage protocols, which compromise fine‐grained spatial fidelity. These limitations are exacerbated in telesurgical and remote consultation scenarios, where bandwidth restrictions necessitate aggressive compression or sub‐sampling, leading to missing viewpoints and degraded visual quality. Addressing these challenges requires novel strategies that can reconstruct anatomically consistent views beyond the original camera perspective, thereby enhancing intraoperative situational awareness and surgical decision‐making.

Our method addresses these challenges by enabling novel view synthesis from sparse laparoscopic inputs, reconstructing anatomically consistent views of regions not directly captured. For example, when a surgeon needs to review an occluded area of the liver surface or visualize a resection margin from a different angle, our approach can generate a realistic novel view without requiring additional physical movement of the camera. This capability is particularly beneficial in cases of limited working space, such as minimally invasive resections of posterosuperior liver segments, where changing the camera position intraoperatively can be challenging or time‐consuming. [3] By reconstructing intermediate and alternative viewpoints, our approach improves intraoperative situational awareness, supports surgical training by generating multiple perspectives from limited recordings, and facilitates postoperative review without the need to store massive amounts of raw video data.

Generating high‐quality intermediate frames from sparsely sampled surgical videos is a challenging yet crucial task in medical video analysis. In minimally invasive procedures such as laparoscopy, the sheer volume of high‐resolution video data poses significant challenges for storage and transmission, often necessitating aggressive compression or frame sub‐sampling [1, 2]. Reconstructing temporally smooth and anatomically accurate sequences from only a few reference frames is essential for downstream applications like surgical navigation and video augmentation. Recent advances in generative modelling [3, 4, 5] have opened new possibilities for sparse‐to‐dense video reconstruction. Latent diffusion models, in particular, have shown strong capabilities in modelling long‐range temporal dependencies within compressed latent spaces [4, 6]. However, most video diffusion approaches rely on calibrated multi‐view inputs or extensive training data, limiting their applicability in surgical environments where annotations and camera parameters are scarce.

Novel view synthesis [7, 8] is a central problem in computer vision, mixed reality, visual effects, and robotics. Techniques such as NeRF [8] and 3D Gaussian splatting [7] achieves remarkable quality from dense multi‐view inputs, but their reliance on abundant calibrated views and high computational costs restricts practical use in clinical or sparse‐view scenarios. Prior works addressing sparse‐view synthesis via regression‐based models [9, 10, 11] typically suffer from limited representational capacity and poor generalization. Recently, diffusion models have enabled zero‐shot novel view synthesis from a single view [12, 13], but remain object‐centric and lack precise camera control. Hybrid methods combining depth‐based warping and diffusion‐based inpainting [14] also frequently produce inconsistent results, especially around occlusions and complex scenes. To address these challenges, the recent method ViewCrafter [3] proposes a solution combining point cloud reconstruction with diffusion models, which partially alleviates these issues. Nevertheless, in practical applications for laparoscopic image synthesis, ViewCrafter still suffers from noticeable artefacts, insufficient colour consistency, and a lack of image detail.

Motivated by these observations, we propose a point‐guided latent diffusion framework combined with a spatial‐transformer enhanced decoder to improve novel view synthesis quality in laparoscopic liver surgery. Our approach requires only two reference images as input to generate content‐consistent intermediate frames without relying on additional information. Specifically, our method starts from an initial point cloud reconstructed from the input reference frames using a dense stereo model and estimates the camera parameters jointly. Regarding camera intrinsic parameters, the principal point is assumed to be at the sensor centre, and the focal length is optimized using the Weiszfeld algorithm [15]. We then apply a next‐best‐view (NBV) [3] based camera trajectory planning algorithm to adaptively generate a sequence of camera poses that effectively reveal occluded regions. The coloured point cloud is rendered along the planned trajectory to produce a coarse sequence of novel‐view frames, which serve as geometric priors and are encoded into the latent space as conditions for our point‐guided latent diffusion model. This model synthesizes high‐quality and content‐consistent intermediate views through iterative denoising. To better preserve anatomical details and local deformations, we design a spatial‐transformer enhanced decoder (STED) that combines residual learning with transformer‐based attention [16]. Importantly, our method does not require camera pose input, significantly enhancing its clinical applicability. Extensive experiments on the P2ILF Challenge dataset [1] demonstrate that our method consistently outperforms popular novel view generation approaches on peak signal‐to‐noise ratio (PSNR) [17], structural similarity index measure (SSIM) [18] and learned perceptual image patch similarity (LPIPS) [19], validating its robustness in detail restoration, motion interpolation and visual consistency with minimal supervision.

This paper is organized as follows: Section 2 is related works, Section 3.3 is our model description, Section 4.3 is experiments and results, and Section 5 is the conclusion.

## 2 Related Works

Novel view synthesis refers to the generation of a range of photorealistic images of a scene from unseen camera viewpoints when limited images with different viewpoints are given. It can be divided into three categories: regression‐based, hybrid‐based and generative‐based approaches. Regression‐based novel view synthesis methods, such as NeRF [8], typically employ multilayer perceptrons (MLPs) architectures establishing implicit 3D representations as neural radiance fields. It constructs the scene representation by MLPs as a function taking position and viewing direction as input and output the RGB colour and volume density. While NeRF shows superiority for generating photorealistic novel views, the requirement of camera pose makes it hard to adapt to the surgical domain. Even if the camera pose can be refined from structure from motion (SfM) using ColorMap, the accuracy of estimation may not be enough for the initialization for 3D Gaussian. Specific approaches, such as multi‐plane representations [9, 10, 11], PixelNeRF [20], or recent real‐time Gaussian splatting methods like PixelSplat [21] and MVSplat [22], have demonstrated effectiveness primarily in category‐specific domains (e.g., human faces and indoor scenes). MIS‐NeRF [23] is proposed recently focused on the minimally‐invasive laparoscopic surgery field where the method aims to alleviate the influences of lighting conditions and specular reflections caused by moving light sources and organ surface. Free‐SurGS [24] introduces an SfM‐free 3D Gaussian splatting for surgical scene reconstruction, which has improved the camera pose estimation by calculating optical flow as the guidance for pose estimation. In the scope of endoscopic video reconstruction, SurgicalGaussian is proposed [25] to address the problem of dynamic scene reconstruction, especially with the existence of tissue deformation. It utilizes the depth and mask of images to initialize 3D Gaussian, where the surgical instrument will be masked out. A pure MLP network is used to predict the offset of dynamic Gaussian over time. Finally, the holes due to the occlusion of mask of instrument are reconstructed smoothly.

Other hybrid methods combine monocular depth estimation with image inpainting for novel view synthesis [26, 27, 28, 29], yet frequently produce visual artefacts due to limited representational capacities, especially in complex medical scenarios (laparoscopic liver surgery), where precise anatomical detail reconstruction is critical. Generative model‐based approaches have explored conditional signals to enhance user control over content generation, such as ControlNet [30] and T2I‐adapter [31]. These strategies have been extended to video generation tasks, incorporating conditions like depth maps [32], trajectories [33], and semantic maps [34]. However, precise camera motion control remains underexplored, with current methods such as AnimateDiff [35], SVD [5], MultiDiff [36], and CameraCtrl [37] providing limited accuracy or generalization capabilities. This limitation is particularly important in laparoscopic liver surgery, where accurate camera pose control and consistent anatomical visualization are the key for effective surgical navigation and decision‐making.

To address these critical issues, our approach leverages explicit 3D point cloud representations in conjunction with latent diffusion models, ensuring precise camera trajectory control and high‐fidelity novel view synthesis tailored specifically for complex and generic surgical scenes encountered in laparoscopic liver surgery.

## 3 Methods

### 3.1 Point‐Guided Video Diffusion Model

**FIGURE 1:**
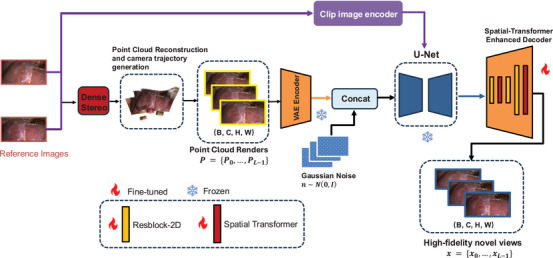
Overview of our method. Given only two reference images of a video, we first build a point cloud representation using dense stereo model DUSt3R, enabling accurate camera movements for free‐viewpoint rendering. To address the large missing regions, geometric distortions and point cloud artefacts in the initial renders, we use a point‐conditioned video diffusion model as an enhanced renderer. A key enhancement in our system is the STED, which fine‐tunes the VAE decoder with spatial transformer modules and 2D residual blocks to improve texture fidelity and colour accuracy.

Our system synthesizes high‐quality novel view sequences from only two reference images of a video by combining point cloud reconstruction with a conditional diffusion model. First, we employ the dense stereo model DUSt3R [38] to reconstruct the point cloud and simultaneously estimate the camera parameters. However, in the view synthesis process, the design of the camera trajectory significantly impacts the synthesis results. Method like LucidDreamer [39] use predefined camera trajectories for scene generation, which overlook the diverse geometry relationships presented in different scenes, resulting in significant occlusions. To effectively reveal occlusions in the view synthesis process and facilitate more complete scene generation, we used a NBV [3] based camera trajectory planning algorithm, which enables the adaptive generation of camera trajectories tailored to handle various scene types. Intermediate camera trajectories are then interpolated to generate a rough sequence of point cloud renders $P=\{P_{0},\cdots ,P_{L-1}\}$. These renders are subsequently encoded into a latent space, producing $\hat{z}=\left\{{\hat{z}}_{0},\cdots ,{\hat{z}}_{L-1}\right\}$. Gaussian noise n∼N(0,I) is added and concatenated with the latent embeddings to form noisy latents. In addition to the latent space condition, the reference images are encoded through a CLIP [40] image encoder, and then combined with the U‐Net via cross‐attention to enhance the model's understanding of the 3D structure. The U‐Net performs iterative denoising to recover clean latent representations, which are then decoded into pixel‐space frames, $x=\{x_{0},\cdots ,x_{L-1}\}$ using a decoder part *D* of variational autoencoder (VAE) [41]. To further the performance of our model, we integrate spatial transformer [42] modules and 2D residual blocks into the VAE decoder. The full architecture is shown in Figure 1.

### 3.2 Spatial‐Transformer Enhanced Decoder Architecture

Video diffusion models have demonstrated strong capabilities in generating structured latent representations z. However, their performance in photorealistic image synthesis and content consistency remains limited. In the context of laparoscopic liver image generation, traditional VAE decoders often produce blurry images with artefacts and fail to preserve high‐frequency details, significantly constraining their clinical applicability. Moreover, due to frequent viewpoint changes caused by laparoscope movement, conventional decoders struggle to recover fine anatomical structures accurately.

To overcome these limitations, we propose an STED to augment the structural representation capacity of latent variables. By integrating spatial transformer blocks, STED effectively restores image details, suppresses artefacts, and enhances the fidelity of high‐frequency textures, thereby improving the quality and realism of synthesized novel views in laparoscopic surgery.

**FIGURE 2:**
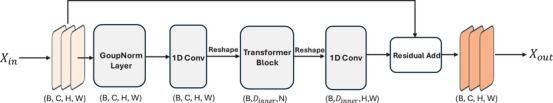
Structure diagram of the spatial transformer. This figure illustrates the detailed structure of the spatial transformer module presented in Figure 1. The input feature map $X_{\text{in}}\in R^{B\times C\times H\times W}$, where B, C, H, and W denote the batch size, number of channels, height, and width, respectively. It is projected to an internal embedding dimension $D_{\text{inner}}=n_{\text{heads}}\times d_{\text{head}}$ for attention‐based spatial modelling. After Transformer processing and residual addition, the output feature map $X_{\text{out}}\in R^{B\times C\times H\times W}$ preserves anatomical fidelity and enhances image realism.

In our STED, we introduce a transformer‐based spatial transformer module [16] explicitly tailored to capture anatomical details specific to laparoscopic liver surgery images. To preserve spatial context and facilitate residual learning, the input feature map $X\in R^{B\times C\times H\times W}$ is first duplicated into a residual branch $X_{\text{in}}$. Next, we apply group normalization (GroupNorm) [43] to stabilize the feature distribution. Following normalization, a 1D convolutional layer projects the feature map into an internal embedding dimension $D_{\text{inner}}=n_{\text{heads}}\times d_{\text{head}}$, yielding a tensor $R^{B\times D_{\text{inner}}\times H\times W}$, ensuring compatibility with subsequent multi‐head attention layers. Here, $n_{\text{heads}}$ denotes the number of attention heads, while $d_{\text{head}}$ represents the dimension of each head. The spatial features are then reshaped into a sequence $X^{\prime }\in R^{B\times N\times D_{\text{inner}}}$, where N=H×W, allowing global interactions among spatial positions to effectively model long‐range dependencies and anatomical relationships crucial for laparoscopic images. Multiple transformer blocks process this sequence, each comprising multi‐head self‐attention, cross‐attention modules [16, 44] and LayerNorm [16]. These collectively enhance spatial feature representation, accurately capturing detailed structures such as liver boundaries, blood vessels and textures. Finally, the processed sequence is reshaped back into its original spatial configuration and projected to the initial channel dimension via another 1D convolutional layer initialized with zero weights, ensuring stable training dynamics. The module concludes by adding this refined spatial representation back to the residual branch $X_{\text{in}}$, preserving essential anatomical details and significantly enhancing the realism of synthesized novel views in laparoscopic scenarios. The full STED architecture is shown in Figure 2.

### 3.3 Spatial‐Transformer Decoder Fine‐Tuning

During the process of fine‐tuning, we adopt a composite loss function inspired by the latent diffusion models (LDM) framework [4]. Specifically, only the decoder of a pretrained VAE is fine‐tuned, while the encoder and quantization layers are kept frozen to maintain the representational integrity of the latent space. The total loss function is formulated as:

(1) $$L_{\mathrm{total}}=\lambda_{\mathrm{rec}}L_{\mathrm{rec}}+\lambda_{\mathrm{perc}}L_{\mathrm{LPIPS}}+\lambda_{\mathrm{KL}}L_{\mathrm{KL}}+\lambda_{\mathrm{adv}}L_{\mathrm{adv}}$$

(2) $$L_{\mathrm{rec}}=\frac{1}{HW}\sum_{i=1}^{H}\sum_{j=1}^{W}{\left(I_{i,j}^{\mathrm{gt}}-I_{i,j}^{\mathrm{gen}}\right)}^{2}$$

(3) $$L_{\mathrm{LPIPS}}=\frac{1}{N}\sum_{i=1}^{N}d\left(\phi \left(x_{i}\right),\phi \left({\hat{x}}_{i}\right)\right)$$

(4) $$L_{\mathrm{KL}}=-\frac{1}{2}\sum_{j=1}^{d}\left(1+\log\left(\sigma_{j}^{2}\right)-\mu_{j}^{2}-\sigma_{j}^{2}\right)$$

(5) $$L_{\mathrm{adv}}=E_{\hat{x}}\left[\mathrm{softplus}\left(-D\left(\hat{x}\right)\right)\right]$$

where the parameters in the reconstruction loss function $L_{\mathrm{rec}}$ in Equation (2) are defined as follows: $I_{i,j}^{\mathrm{gt}}$ and $I_{i,j}^{\mathrm{gen}}$ represent the ground truth and generated pixel values at location (i,j), respectively, H and W are the height and width of the image, and N=H×W is the total number of pixels. In Equation (3) $x_{i}$ and ${\hat{x}}_{i}$ represent the ith pixel of the ground truth and reconstructed images, respectively. The function ϕ(·) denotes a deep feature extractor, such as a pretrained VGG [45] or AlexNet network [46], while d(·,·) computes the distance between deep feature vectors. For the KL divergence loss in Equation (4), $\mu_{j}$ and $\sigma_{j}$ are the mean and standard deviation of the jth dimension of the latent variable, and d is the dimension of the latent space. For the adversarial loss in Equation (5), D(·) denotes the output of a patch‐based discriminator for a reconstructed image, and $\mathrm{softplus}\left(a\right)=\log\left(1+e^{a}\right)$. The operator $E_{\hat{x}}\left[\cdot \right]$ indicates the expectation over reconstructed images. The weighting coefficients $\lambda_{\mathrm{rec}},\lambda_{\mathrm{perc}},\lambda_{\mathrm{KL}},\lambda_{\mathrm{adv}}$ are hyperparameters that control the relative contributions of the reconstruction, perceptual, KL regularization, and adversarial loss terms. The impact of these hyperparameters is further analysed through ablation studies in Section 4.3.

## 4 Experiments and Results

### 4.1 Experimental Setup

#### 4.1.1 Datasets Preparation

We employ a dual‐source image dataset comprising 3000 images (576×1024 resolution) extracted from laparoscopic surgery videos of 10 patients used in the P2ILF challenges [1] and 12,000 images of the same resolution selected from the large‐scale YouHQ [47] video dataset. Clinical liver images serve as primary training data to capture domain‐specific anatomical and semantic features, while diverse natural scenes from YouHQ are utilized for decoder fine‐tuning. This strategy enhances the model's ability to synthesize visually realistic outputs under varied textures and lighting conditions, thereby significantly improving the quality and generalization of the generated images.

#### 4.1.2 Implementation Details

We conducted our experiments on a single NVIDIA L40S GPU. Following the training setup of latent diffusion models (LDM) [4], both the autoencoder and discriminator used a learning rate of $1\times 10^{-4}$, which balances stable convergence and effective gradient updates in adversarial training. A batch size of 12 was adopted, as a compromise between GPU memory constraints and sufficient gradient diversity, consistent with the practices in LDM. The model was trained for 350 epochs until convergence, following the fine‐tuning schedule recommended for VAEs in the LDM framework, which avoids premature stopping while preventing overfitting. Mixed precision training was employed to accelerate computation and reduce memory usage, again in line with LDM's design choices. During training, only the decoder of the pretrained VAE was fine‐tuned, while the encoder and its quantization layers were kept frozen. This design mirrors the LDM strategy of preserving the representational capacity of the pretrained latent space while adapting the decoder to the downstream task.

### 4.2 Results and Discussion

#### 4.2.1 Quantitative Results

**FIGURE 3:**
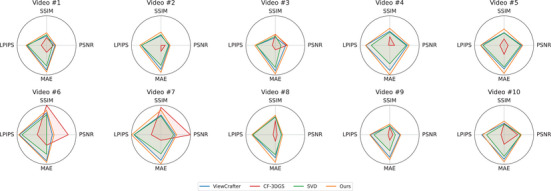
Radar charts comparing the perceptual metrics of different methods across 10 liver laparoscopic videos. Higher values indicate better performance, with LPIPS and MAE metrics inverted for consistency.

In Table 1, we quantitatively compare our method against ViewCrafter [3], CF‐3DGS [48], and SVD [5], using PSNR) [17], SSIM [18], LPIPS [19] and mean absolute error (MAE), which is defined as the average of the absolute differences between the reconstructed image and the ground truth at the pixel level) [49]. The MAE is evaluated across ten representative laparoscopic liver surgery scenes selected from the P2ILF video dataset. Our method consistently achieves superior performance across these indicators, showing higher PSNR and SSIM values, as well as lower LPIPS scores and MAE. For example, in Vid #4, our method outperforms ViewCrafter by 0.46 dB in PSNR, improves SSIM by 2.46%, and slightly reduces LPIPS from 0.193 to 0.190. In Vid #7, we observe a significant improvement in LPIPS by approximately 17.8%, along with an increase of 0.38 dB in PSNR and a nearly 4.0% enhancement in SSIM. While ViewCrafter maintains a generally balanced trade‐off between sharpness and structure, its performance is slightly inferior to ours across most scenes. SVD, on the other hand, exhibits competitive LPIPS scores and strong temporal smoothness, but falls short in structural fidelity and sharpness, as indicated by relatively lower PSNR and SSIM values, particularly in Vid #6 and Vid #10. Notably, CF‐3DGS performs exceptionally well in terms of PSNR and SSIM in Vid #6 and Vid #7, achieving peak PSNR values of 23.836 and 25.799 dB, respectively. However, despite these high‐fidelity signals, CF‐3DGS struggles with perceptual consistency. Its LPIPS scores in these scenes are substantially higher than those of our model: 0.362 versus 0.147 in Vid #6, and 0.358 versus 0.129 in Vid #7, suggesting limited perceptual realism under dynamic viewpoints. Moreover, our method consistently achieves the lowest MAE values across all scenes (e.g., 0.060 in Vid #4 and 0.061 in Vid #7), indicating higher pixel‐wise accuracy and reduced reconstruction error compared to the other baselines. Figure 3 presents radar charts visualizing the performance of four methods—ViewCrafter, CF‐3DGS, SVD, and our model—across 10 liver laparoscopic video sequences. Each chart summarizes four perceptual metrics: PSNR, SSIM, LPIPS and MAE. To ensure fair visual comparison, all metrics are normalized, with LPIPS and MAE inverted as lower values indicate better performance. The radar plots highlight the consistent superiority of our model in both reconstruction quality and perceptual fidelity across most videos.

**TABLE 1:** Quantitative comparison of PSNR, SSIM, LPIPS and MAE between ViewCrafter, CF‐3DGS, SVD, and our model in 10 liver laparoscopic videos.

**Video ID**	**ViewCrafter**				**CF‐3DGS**				**SVD**				**Ours**			
	PSNR↑	SSIM↑	LPIPS↓	MAE↓	PSNR↑	SSIM↑	LPIPS↓	MAE↓	PSNR↑	SSIM↑	LPIPS↓	MAE↓	PSNR↑	SSIM↑	LPIPS↓	MAE↓
Vid #1	20.542	0.743	0.240	0.070	20.149	0.732	0.408	0.105	19.931	0.751	0.251	0.078	**20.743**	**0.763**	**0.232**	**0.066**
Vid #2	20.493	0.745	0.243	0.071	19.473	0.681	0.470	0.107	20.331	0.751	0.246	0.079	**20.726**	**0.768**	**0.223**	**0.065**
Vid #3	21.027	0.732	0.252	0.068	21.289	0.754	0.435	0.110	19.830	0.741	0.250	0.075	**21.527**	**0.753**	**0.232**	**0.062**
Vid #4	22.831	0.773	0.193	0.069	19.627	0.738	0.457	0.119	22.560	0.767	0.256	0.082	**23.291**	**0.792**	**0.190**	**0.060**
Vid #5	22.846	0.764	0.200	0.072	19.435	0.723	0.421	0.101	22.554	0.762	0.226	0.076	**23.076**	**0.788**	**0.197**	**0.063**
Vid #6	19.870	0.825	0.155	0.067	**23.836**	**0.874**	0.362	0.098	18.431	0.812	0.185	0.080	20.313	0.841	**0.147**	**0.064**
Vid #7	21.986	0.810	0.157	0.068	**25.799**	**0.860**	0.358	0.108	21.599	0.803	0.160	0.081	22.366	0.842	**0.129**	**0.061**
Vid #8	19.929	0.796	0.208	0.073	18.877	0.773	0.444	0.106	19.987	0.799	0.211	0.079	**20.234**	**0.808**	**0.199**	**0.064**
Vid #9	20.968	0.738	0.246	0.074	19.224	0.730	0.451	0.108	19.844	0.742	0.288	0.087	**21.178**	**0.751**	**0.239**	**0.069**
Vid #10	22.531	0.756	0.211	0.070	21.823	0.753	0.433	0.100	22.642	0.749	0.267	0.076	**22.746**	**0.770**	**0.220**	**0.065**

Table 2 provides the average performance across all ten evaluated scenes, further highlighting our model's superior effectiveness. Compared to ViewCrafter, our method demonstrates an average increase of 0.517 dB in PSNR, an improvement of 2.2% in SSIM, and a notable reduction of 7.7% in LPIPS. Similarly, compared to CF‐3DGS, our model achieves a 0.867 dB gain in PSNR, a 2.1% increase in SSIM, and a substantial 20.8% reduction in LPIPS, highlighting its advantage in perceptual fidelity. In contrast to SVD, our approach yields a 0.949 dB improvement in PSNR, a 1.0% gain in SSIM, and a 12.8% decrease in LPIPS. In terms of MAE, our method attains the lowest mean error of 0.0639, outperforming ViewCrafter (0.0702), CF‐3DGS (0.1062), and SVD (0.0793), further demonstrating its robustness and reliability in pixel‐level reconstruction. These consistent improvements across all metrics underscore the potential of our approach to generate structurally faithful and perceptually realistic novel views, which is especially critical for clinical applications requiring high anatomical accuracy and visual coherence.

**TABLE 2:** Average (± std) PSNR, SSIM, LPIPS and MAE across 10 liver laparoscopic videos.

Method	PSNR↑	SSIM↑	LPIPS↓	MAE↓
ViewCrafter	21.099±0.970	0.768±0.030	0.214±0.033	0.070±0.002
CF‐3DGS	20.854±1.670	0.759±0.055	0.410±0.039	0.106±0.006
SVD	20.525±1.480	0.763±0.032	0.244±0.034	0.079±0.003
**Ours**	**21.835** ± **0.950**	**0.783** ± **0.031**	**0.202** ± **0.028**	**0.064** ± **0.003**

#### 4.2.2 Qualitative Results

**FIGURE 4:**
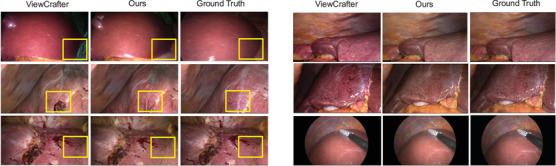
Qualitative comparison between our method and ViewCrafter. The left column illustrates that our novel view synthesis produces significantly lower and less identifiable artifacts. The right column indicates that our method preserves colours that are perceptually closer to the ground truth.

**FIGURE 5:**
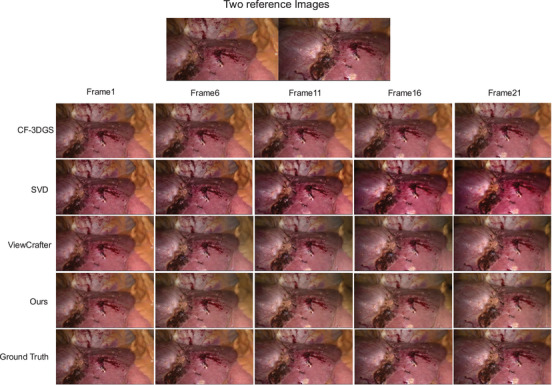
Synthesis results from SVD, CF‐3DGS, ViewCrafter, ours, and ground truth. To evaluate temporal consistency and visual quality, we sample one frame every five frames from a video sequence starting at the first frame. Compared to SVD and ViewCrafter, our method shows better colour stability, while compared to CF‐3DGS, it achieves higher spatio‐temporal coherence. The quantitative analysis of the above frame sequence can be found in Table 3.

In addition to delivering superior quantitative results, our method also demonstrates clear advantages in visual quality, as illustrated by the qualitative comparisons in Figure 4. Specifically, our approach produces novel views with fewer artefacts and colour renderings that are more consistent with the ground truth, compared to ViewCrafter. Beyond this, Figure 5 presents qualitative reconstruction results on the P2ILF dataset, comparing SVD, CF‐3DGS, ViewCrafter, our method, and ground truth. For a more detailed evaluation of temporal consistency, our method samples five frames from the video sequence, with a frame interval of five, and compares the visual results. Among all approaches, our method achieves the most realistic colour rendering and the fewest visual artifacts, closely resembling the ground truth images. Note that CF‐3DGS approach suffers from a limited range of novel view synthesis compared with those generative‐based approaches, suggesting that the potential of generative models for novel view generation when reference images are scarce. These results highlight the effectiveness of our model in generating perceptually faithful novel views in laparoscopic videos, while maintaining temporal consistency and visual quality across the sampled frames.[Table htl270032-tbl-0003]

### 4.3 Ablation Study

To evaluate the contribution of each loss component in our VAE fine‐tuning process, we conducted a series of ablation experiments. Our baseline model employs the loss function introduced in Section 3.3. In our experiments, the loss weights were selected based on prior ablation studies to balance image fidelity, perceptual quality and training stability. A small KL divergence weight ($1\;\times 10^{-6}$) follows Kingma and Welling [50], avoiding over‐regularization that can degrade generation quality. The pixel‐wise reconstruction loss is set to 0.1, as it remains essential for fidelity [51]. The discriminator loss weight of 0.3 helps stabilize GAN training [52]. Perceptual loss (LPIPS) is weighted at 0.1 [19, 53], enhancing visual realism without overwhelming pixel accuracy.

To isolate the effect of each loss component, we systematically removed individual terms from the full loss function. Table 4 summarizes the experimental configurations and their performance metrics. Our ablation results show that each loss component plays a complementary role in optimizing both objectives and perceptual qualities, and the best VAE fine‐tuning performance is achieved when all loss terms are balanced. In addition to evaluating the effects of different loss components, we conducted an ablation study to assess the architectural improvements of our VAE model. In particular, we analysed the impact of integrating a spatial attention mechanism, implemented via a spatial‐transformer (ST), and decoder fine‐tuning (FT). This module enables the model to adaptively focus on salient spatial regions during both encoding and decoding stages. The experimental results summarized in Table 5 demonstrate the contribution of these architectural components to perceptual and pixel‐level quality improvements.

**TABLE 3 htl270032-tbl-0003:** Quantitative evaluation of synthesis results from SVD, CF‐3DGS, ViewCrafter, and ours. To assess temporal consistency and visual quality, one frame is sampled every five frames from a video sequence starting at the first frame.

**Method**	**Frame 1**			**Frame 6**			**Frame 11**			**Frame 16**			**Frame 21**		
	PSNR↑	SSIM↑	LPIPS↓	PSNR↑	SSIM↑	LPIPS↓	PSNR↑	SSIM↑	LPIPS↓	PSNR↑	SSIM↑	LPIPS↓	PSNR↑	SSIM↑	LPIPS↓
ViewCrafter	22.763	0.740	0.215	18.416	0.721	0.276	18.907	0.747	0.251	20.004	0.747	0.254	22.008	0.756	0.228
CF‐3DGS	14.976	0.690	0.281	15.230	0.706	0.270	16.395	0.745	0.472	17.405	0.741	0.562	19.079	0.772	0.501
SVD	22.297	0.753	0.264	18.457	0.724	0.301	18.692	0.741	**0.237**	19.451	0.737	0.263	21.401	0.770	**0.171**
Ours	**22.859**	**0.756**	**0.209**	**18.714**	**0.738**	**0.269**	**19.105**	**0.758**	0.241	**20.039**	**0.752**	**0.253**	**22.134**	**0.774**	0.201

**TABLE 4:** Ablation study on the effect of different loss term weights ($\lambda_{\text{rec}},\lambda_{\text{perc}},\lambda_{\text{KL}},\lambda_{\text{adv}}$) for VAE decoder fine‐tuning. Performance is reported in terms of PSNR [17], SSIM [18] and LPIPS [19].

ID	$\lambda_{\text{rec}}$	$\lambda_{\text{perc}}$	$\lambda_{\text{KL}}$	$\lambda_{\text{adv}}$	PSNR↑	SSIM↑	LPIPS↓
Baseline	0.1	0.1	$10^{-6}$	0.3	**22.746**	**0.770**	**0.211**
A	0	0.1	$10^{-6}$	0.3	22.531	0.755	0.221
B	0.1	0	$10^{-6}$	0.3	22.695	0.778	0.231
C	0.1	0.1	0	0.3	22.699	0.778	0.230
D	0.1	0.1	$10^{-6}$	0	22.734	0.770	0.221

**TABLE 5:** Ablation study evaluating the impact of the spatial transformer (ST) module and decoder fine‐tuning (FT) on reconstruction performance. Metrics reported: PSNR, SSIM, and LPIPS.

Model variant	PSNR↑	SSIM↑	LPIPS↓
Baseline (w/o ST, w/o decoder FT)	22.536	0.752	0.241
Baseline (w/o ST, + decoder FT)	22.631	0.759	0.223
Baseline (+ ST, + decoder FT)	**22.746**	**0.770**	**0.211**

## 5 Discussion and Conclusion

### 5.1 Limitations and Failure Cases

**FIGURE 6:**
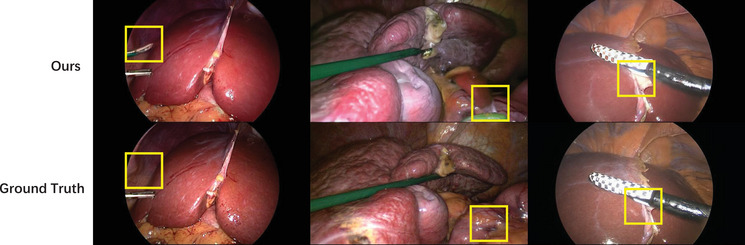
Failure cases in laparoscopic liver surgery, including rapid instrument motion and large camera viewpoint changes, which lead to artefacts, tearing, and colour inconsistency in the reconstructed views.

While our method demonstrates strong performance on laparoscopic liver surgery videos, its robustness is reduced in scenarios with extreme scene dynamics. As shown in Figure 6, rapid surgical instrument motion can introduce motion discontinuities that are difficult to model from sparse frames, resulting in ghosting and tearing artefacts in the synthesized views. Similarly, large inter‐view camera shifts exacerbate misalignment between the coarse geometric prior and the image evidence, leading to noticeable colour inconsistencies and residual artifacts near occlusion boundaries. These limitations are partly due to the reliance on static point cloud priors and the absence of explicit motion compensation in our pipeline. Future work will explore integrating robust motion modelling, occlusion‐aware consistency constraints, and multi‐organ training to improve adaptability across diverse surgical scenes. In addition, our current implementation relies on a high‐end NVIDIA L40S GPU for both training and inference, which may limit direct applicability in typical clinical environments. This dependency arises from the computational demands of latent diffusion models and high‐resolution rendering. To address this, future work will explore model compression, mixed‐precision inference, and deployment‐oriented optimizations such as TensorRT acceleration and knowledge distillation, enabling real‐time performance on more accessible hardware.

### 5.2 Conclusion and Future Work

We have presented a point‐guided latent diffusion framework with a spatial‐transformer enhanced decoder for novel view synthesis in laparoscopic liver surgery. Our method synthesizes high‐quality intermediate frames from sparse inputs without the need for camera pose, and achieves superior visual fidelity and structural consistency over existing approaches. To better capture the spatial‐temporal relationship and preserve the anatomical details, we also introduce spatial‐transformer enhanced decoder, which is connected after denoising U‐Net to synthesis high‐fidelity novel views. The ablation study reveals that this module can improve the quality for the generated novel views. The method is evaluated on the P2ILF video dataset. Our evaluation results demonstrate that the proposed model leads the performance of novel synthesis compared with SVD, CF‐3DGS and ViewCrafter in terms of PNSR, SSIM and LPIPS metrics. Future work will focus on extending the approach to dynamic surgical scenes and improving generalization to challenging anatomical cases.

## Author Contributions

**Wenzhe Tang**: conceptualization, data curation, formal analysis, investigation, methodology, software, validation, visualization, writing – original draft, writing – review and editing. **Tao Chen**: data curation, investigation, methodology, software, supervision, validation, visualization, writing – original draft, writing – review and editing. **Yamid Espinel**: data curation, writing – review and editing. **Shahid Farid**: data curation, writing – review and editing. **Emmanuel BUC**: data curation, resources, writing – review and editing. **Adrien Bartoli**: data curation, resources, writing – review and editing. **Sharib Ali**: conceptualization, formal analysis, investigation, methodology, project administration, resources, supervision, validation, writing – original draft, writing – review and editing.

## Conflicts of Interest

The authors declare no conflicts of interest.

## Data Availability

The authors have nothing to report.
